# Molecular profiling of breast cancer in native American women reveals distinct genomic and transcriptomic features

**DOI:** 10.1038/s41698-026-01373-6

**Published:** 2026-03-17

**Authors:** Fangfang Guo, Laurie E. Littlepage, M. Sharon Stack, Jun Li

**Affiliations:** 1 Department of Applied and Computational Mathematics and Statistics, University of Notre Dame, Notre Dame, IN USA; 2 Harper Cancer Research Institute, University of Notre Dame, Notre Dame, IN USA; 3 Department of Chemistry and Biochemistry, University of Notre Dame, Notre Dame, IN USA

**Keywords:** Biomarkers, Cancer, Computational biology and bioinformatics, Genetics, Oncology

## Abstract

Breast cancer outcomes vary across populations, yet Native American women remain scarcely represented in tumor-genomic resources, limiting population-specific molecular insights. We generated matched somatic mutation, copy-number, and RNA-seq profiles for 17 breast tumors from Native American women and performed race-stratified comparisons with White cases from The Cancer Genome Atlas (TCGA) Breast Invasive Carcinoma (BRCA) cohort (TCGA-BRCA). We observed population-associated differences across molecular layers, including higher mutation frequencies in *ARID1B*, *NOTCH4*, and MHC class II genes (*HLA-DRB1*/*HLA-DRB5*) in Native American tumors, and broader CNV alterations in White tumors. Integrative analyses highlighted antigen processing/presentation and cell-adhesion pathways, with class II alterations in Native American tumors and class I gains (e.g., *HLA-A*/*HLA-B*) plus *CD274* amplification in White tumors, suggesting differences in immune visibility and checkpoint modulation. We also noted contrasts in nucleotide-excision-repair involvement (*ERCC5*/*POLE* mutations vs *ERCC1*/*CUL4A* CNV gains), and mutational-signature analysis indicated greater MMR- and AID/*POLE*-associated exposures in the White cohort. To our knowledge, this study provides an initial multi-omics characterization of breast tumors from Native American women and offers a resource and hypotheses for larger, harmonized studies to assess prognostic and therapeutic relevance.

## Introduction

Breast cancer remains the most common cancer among women worldwide and a leading cause of cancer-related mortality^1^. Although advances in screening, molecular subtyping, and targeted therapies have improved overall outcomes, survival gains have not been equitably realized across populations^2^. Emerging evidence suggests that population-specific molecular mechanisms contribute to these differences. For instance, Parada et al.^3^ reported elevated expression of poor-prognosis genes, such as *PSPH* and *TYMS* among Black women, strongly associated with recurrence risk. Huo et al.^4^ showed that, within the basal-like/TNBC subtype, African-ancestry women experience worse survival. Moreover, Thorn et al.^5^ found that non-European patients-especially those of African descent-are more likely to present with aggressive tumor subtypes and experience earlier mortality. These disparities underscore the need for population-specific molecular characterization to uncover disease-relevant molecular drivers and ensure equitable benefits of precision oncology.

Although extensive, breast cancer genomics resources that underpin current knowledge remain heavily weighted toward patients of European ancestry and include almost no Native American women. In The Cancer Genome Atlas (TCGA) Breast Invasive Carcinoma (BRCA) cohort (TCGA-BRCA, *n* = 1098), only a single case is recorded as American Indian/Alaska Native (AI/AN); White cases constitute the majority (*n* = 757). (Throughout, we use “Native American” for individuals categorized as AI/AN in U.S. datasets, retaining “AI/AN” only when quoting source labels; we use “White” to refer to the TCGA race category “White”.) This paucity of AI/AN tumor data hampers the understanding of breast cancer biology in Native American women and forces treatment decisions to rely on evidence derived from other populations. The concern is heightened by reports of higher cancer burdens in some regions of the United States, including more advanced disease at diagnosis^6^ and worse survival^7^. Molecular studies are therefore needed to reveal Native American-specific pathways related to tumor development, immune escape, and treatment response that would otherwise go unrecognized.

To date, relatively few studies have interrogated breast tumors from Native American patients, and most have been constrained in scope or assay modality. Germline-focused work has identified population-specific variants; Liede et al.^8^ reported a shared *BRCA1* alteration in Cree and Ojibwe families consistent with a founder event, while Dobbin et al.^9^ observed several *BRCA2* variants at elevated frequencies in Amazonian Amerindian populations, informing hereditary risk but not tumor-intrinsic alterations. Boone et al.^10^ analyzed inherited *CYP19A1* variants and found ancestry-associated risk signals without validation in tumors. At the protein level, Kaur et al.^11^ used immunohistochemistry (IHC) to assess ER, PR, HER2, Ki-67 (MIB-1), and other markers in AI/AN cases from different regions, noting regional differences (e.g., p53, EGFR, HER2), and Marker et al.^12^ showed that Indigenous American genetic ancestry is positively associated with HER2-positive breast cancer in Latin American women. Clinical-utilization data also indicate that AI/AN women are less likely to receive recurrence-score testing and, when tested, more often have high-risk scores^13^. Collectively, these efforts provide useful context but stop short of comprehensive tumor genomics or transcriptomics at scale; Native American patients remain underrepresented in large sequencing resources. To our knowledge, no published study has reported tumor multi-omics profiling of breast cancer in Native American women.

Here, we address this gap by generating matched somatic mutation, copy number variation (CNV), and RNA-seq profiles for 17 breast tumors from Native American women. Using race-stratified comparisons with White patients from TCGA, we conduct a multi-omics analysis that identifies population-associated differences in mutational spectra, recurrent CNV events, and gene-expression programs. Integrative pathway analyses further suggest distinct patterns of immune regulation, DNA repair processes, and oncogenic signaling between Native American and White tumors. By moving beyond germline association and IHC-based studies, this study provides an initial multi-omics characterization of Native American breast tumors and generates hypotheses that may inform future biomarker development and therapeutic research for this historically understudied population.

## Results

### Cohort and data generation overview

We analyzed tumor tissues from 17 Native American women and generated matched multi-omics profiles for each case: targeted DNA sequencing (648 genes) with panel-derived copy-number calls and whole-transcriptome RNA-seq (Methods). Matched normal DNA supported somatic variant calling (mean depths ~500 × tumor/150 × normal), variants were harmonized to MAF format, and copy-number alterations were initially obtained at the segment level from the targeted panel. As a comparator, we used White cases from TCGA-BRCA with available data for each modality (somatic mutation: *n* = 689; CNV: *n* = 727; RNA-seq: *n* = 754; Methods). TCGA modality counts reflect samples with available data and are not necessarily the same individuals across mutation, CNV, and RNA-seq. To mitigate cross-platform effects, mutation and CNV analyses were restricted to the 648 panel genes, and RNA-seq analyses used rank-based normalization across samples (Methods). We report our analyses in the following order: (i) gene-level somatic mutation differences; (ii) differential expression; (iii) copy-number differences; (iv) mutational signatures; and (v) integrative pathway analyses across data layers.

### DNA Mutation Analysis

**Fig. 1:**
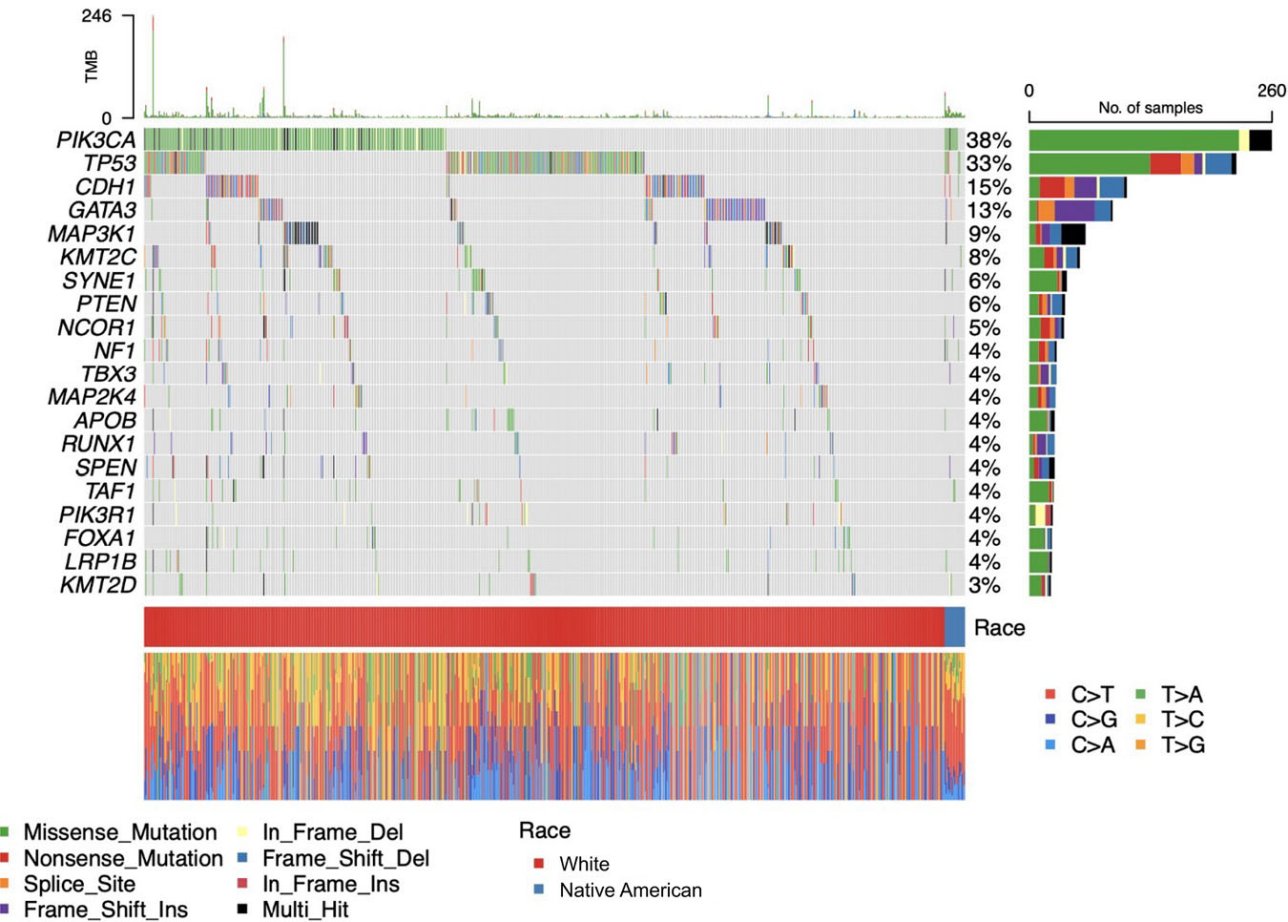
Oncoplot illustrating the top 20 most frequently mutated genes in 17 Native American (blue bar) and 689 White (red bar) samples. Genes are ranked by mutation frequency within and across populations. Each column corresponds to a sample, and each row represents a gene. The color scheme within the heatmap denotes distinct mutation types, including missense mutations (green), nonsense mutations (red), and additional categories as detailed in the legend. The bar plot on the right shows the number of samples with mutations in each gene. The bottom bar summarizes the distribution of mutation types in individual samples.

Figure 1 displays the 20 most frequently mutated genes in the combined cohort of both populations, based on somatic mutations in the 648-gene panel. Each column represents a sample, and each row corresponds to a gene. The colored blocks in the heatmap show different types of mutations. The bar plot on the right shows how many samples had mutations in each gene, while the horizontal color bar below the heatmap indicates the race of each sample (red for White, blue for Native American).

From this plot, we see that *PIK3CA* and *TP53* are the most commonly mutated genes in the two groups, with mutation frequencies of 38% and 33%, respectively. These findings are consistent with previous reports identifying them as key driver genes that are mutated in breast cancer^14,15^. *PIK3CA* mutations are typically activating and are often missense mutations that enhance PI3K signaling, thereby promoting tumor growth and survival^16^. *TP53* mutations, in contrast, are usually inactivating and can include nonsense, frameshift, and splice-site mutations, leading to loss of tumor suppressor function^17^. These mutation patterns are visualized in the bar plot on the right side of Fig. 1.

Other frequently mutated genes include *CDH1* and *GATA3*, which are also known to play important roles in breast cancer. *CDH1*, a tumor suppressor gene involved in cell adhesion, is commonly inactivated in lobular breast cancer, and its loss is associated with enhanced invasiveness and metastatic potential^18^. *GATA3*, a transcription factor crucial for luminal cell differentiation, is frequently mutated in luminal A tumors^14^, driving altered transcriptional programs and tumor progression^19–21^ and influencing estrogen receptor-mediated transcriptional response^22^.

**Fig. 2:**
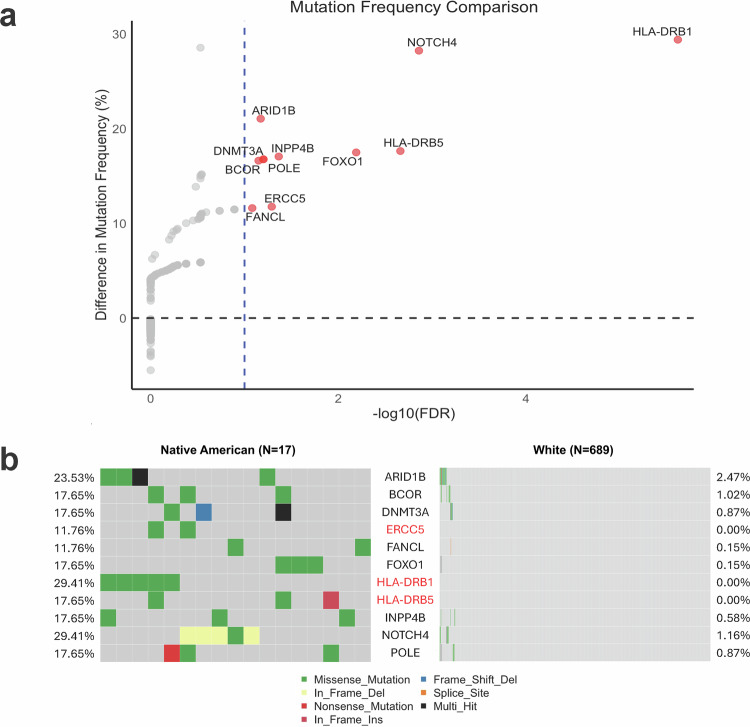
Race-associated differences in somatic mutation patterns between Native American and White breast cancer patients. **a** Mutation frequency comparison between Native American and White populations. Each point represents a gene. The x-axis shows $-{\log }_{10}(\,{\rm{FDR}})$ from Fisher’s exact test, and the y-axis shows the difference in mutation frequency (Native American % - White %). Genes with FDR < 0.1 are highlighted in red. Positive values on the y-axis indicate higher mutation frequencies in Native American patients. **b** Somatic mutation patterns for 11 genes that showed significantly different mutation frequencies between Native American and White breast cancer patients. Each row represents one gene, and each column represents one patient sample. The left panel displays Native American samples (*N* = 17), and the right panel shows White samples (*N* = 689). The percentages on the left and right sides indicate the proportion of mutated samples in each population for each gene. The color scheme within the heatmap indicates the presence and type of mutation in a given sample. Gray indicates no mutation in that sample. Genes highlighted in red were mutated exclusively in Native American samples and not observed in any White samples.

To identify genes with differing mutation frequencies between populations, we performed Fisher’s exact test for each gene and adjusted *p*-values using the Benjamini-Hochberg (BH) method^23^ to control false discovery rate (FDR). Figure 2a summarizes the results of this comparison. Each point represents a gene, with the x-axis showing the statistical significance of the test (plotted as $-{\log }_{10}$(FDR)), and the y-axis showing the difference in mutation frequency between Native American and White cohorts. Genes with positive y-axis values are more frequently mutated in Native American samples, while negative values would indicate higher frequencies in White samples. Genes shown in red passed the significance threshold (FDR < 0.1), indicating that their mutation frequencies differ significantly between the two populations.

Based on this analysis, 11 genes were identified as having significantly higher mutation frequencies in Native American patients, while no genes were significantly more mutated in White patients. These include *ARID1B*, *NOTCH4*, *HLA-DRB1*, and *HLA-DRB5*, among others. *ARID1B* is a chromatin remodeler frequently altered in cancers^24^. *NOTCH4* is part of the Notch signaling pathway involved in cell fate decisions^25^. *HLA-DRB1* and *HLA-DRB5* are immune-related genes critical for antigen presentation^26^. These suggest that both regulatory and immune processes may be impacted by the mutations identified in the Native American cohort. Detailed statistical results for the 11 differentially mutated genes are provided in Supplementary Data 1.

Figure 2b provides a detailed view of the mutation types and frequencies for these 11 genes across the two populations. Each column represents a sample, and each row corresponds to one of the genes. The left panel displays Native American samples, and the right panel shows White samples. Different mutation types are color-coded as indicated in the legend. From this plot, we observe that *ARID1B* is much more frequently mutated in Native American samples (23.53%) compared to White samples (2.47%). In addition, genes marked in red in the plot, including *ERCC5*, *HLA-DRB1*, and *HLA-DRB5*, are mutated only in Native American samples. Furthermore, specific mutation types vary by gene; for instance, in-frame deletions in *NOTCH4* are uniquely observed in Native American patients.

### RNA-seq Differential Expression Analysis

**Fig. 3:**
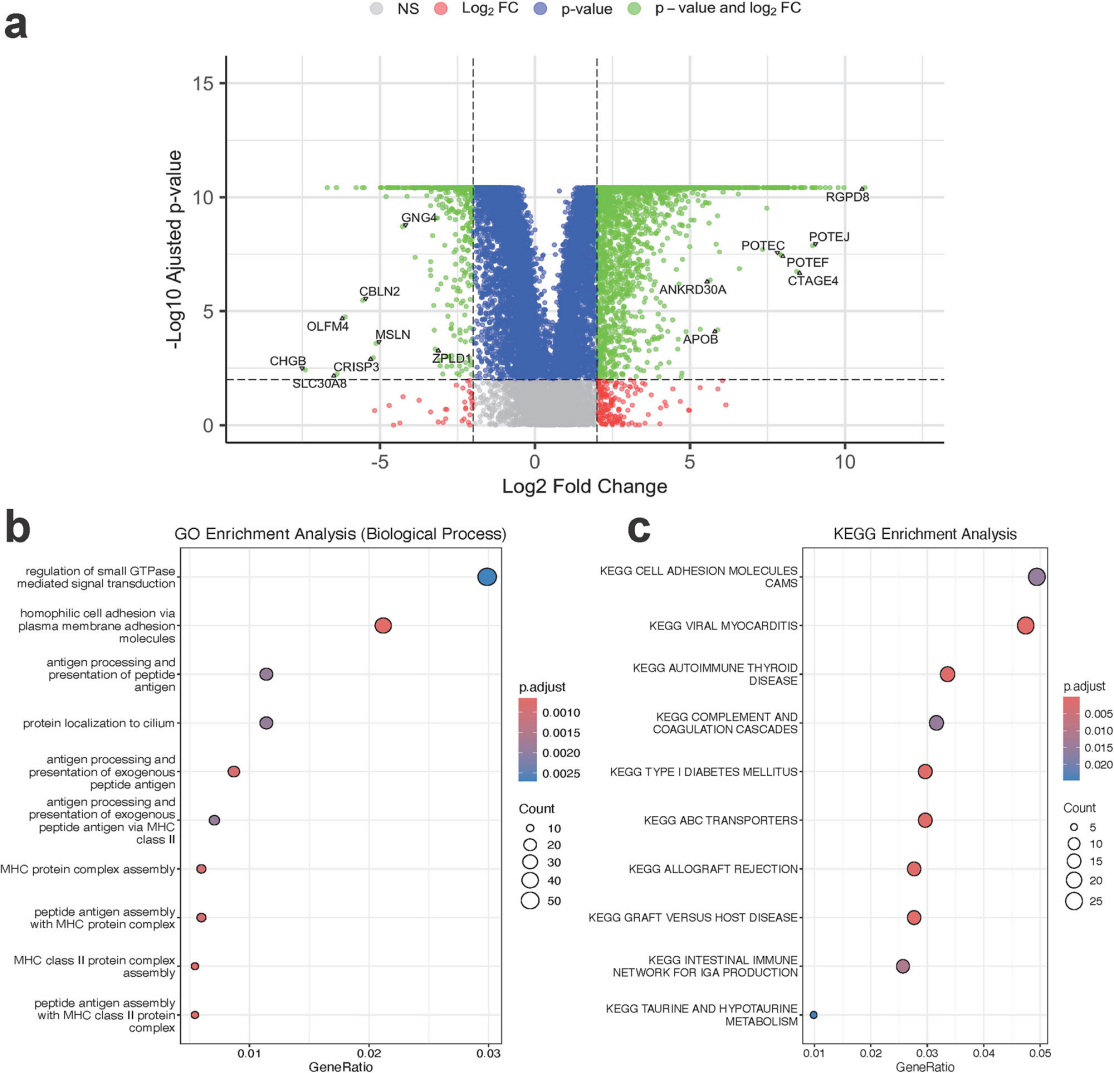
Differential expression analysis between Native American and White patients. **a** Volcano plot. The x-axis represents the log2 fold change (Log2FC; computed on normalized expression), with positive values indicating genes with higher expression in Native American patients. The y-axis represents the $-{\log }_{10}$ adjusted *p*-value. Genes highlighted in green meet the significance thresholds for both *p*-value and Log2FC, while red and blue points indicate genes significant for Log2FC or *p*-value alone, respectively. Non-significant genes are shown in gray. **b**, **c** GO and KEGG functional enrichment of DEGs. Dot size represents the number of DEGs in each pathway or process, and color intensity reflects statistical significance (adjusted *p*-value).

RNA-seq analysis identified 2293 differentially expressed genes (DEGs) between Native American and White breast cancer patients, based on an adjusted *p*-value < 0.01 and an absolute log_2_ fold change ≥ 2. These DEGs are visualized in a volcano plot (Fig. 3a), where each point represents a gene, with the x-axis showing the log_2_ fold change (positive values indicate higher expression in Native American patients) and the y-axis showing the statistical significance ($-{\log }_{10}$ adjusted *p*-value). Genes highlighted in green meet both thresholds. From this plot, we observed that genes, such as *RGPD8*, *POTEJ*, and *CTAGE4* were significantly upregulated in Native American patients, while genes, such as *OLFM4*, *CHGB*, and *GNG4* were significantly downregulated. The full statistical results for these DEGs are provided in Supplementary Data 3.

To understand the potential functional impact of these expression differences, we performed pathway enrichment analysis using GO and KEGG databases (Fig. 3b, c). In these bubble plots, each point represents an enriched biological process or pathway. The x-axis indicates the proportion of DEGs involved (GeneRatio), the dot size shows the number of genes in each pathway, and the color reflects the adjusted *p*-value. From the GO analysis, we saw enrichment in immune-related processes, such as regulation of small GTPase-mediated signaling, antigen processing and presentation, and MHC protein complex assembly. The KEGG analysis highlighted pathways, such as cell adhesion molecules (CAMs) and complement and coagulation cascades, which are important in immune response and cell-cell communication^27,28^. These findings suggest that differences in tumor immune activity and signaling pathways may contribute to population-specific biological characteristics between Native American and White patients.

### CNV Analysis

**Fig. 4:**
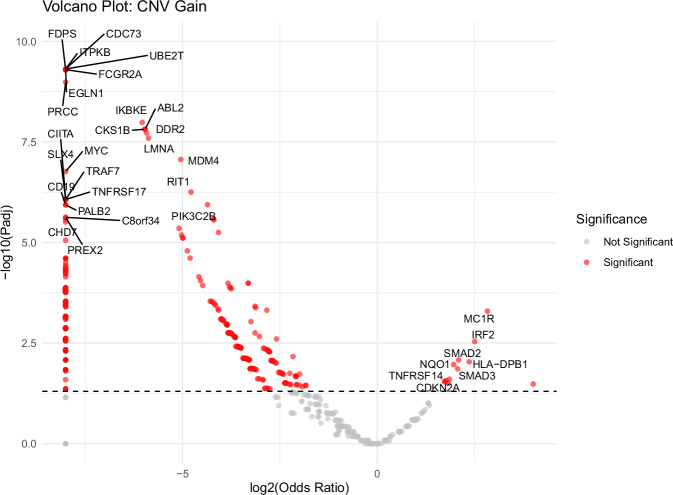
Volcano plot showing differences in copy number gains between Native American and White cohorts. The x-axis represents ${\log }_{2}$(Odds Ratio), where negative values indicate higher CNV frequencies in White samples, and positive values indicate higher frequencies in Native American samples. Red dots denote genes with significantly different CNV gain frequencies (adjusted *p*-value < 0.05).

**Fig. 5:**
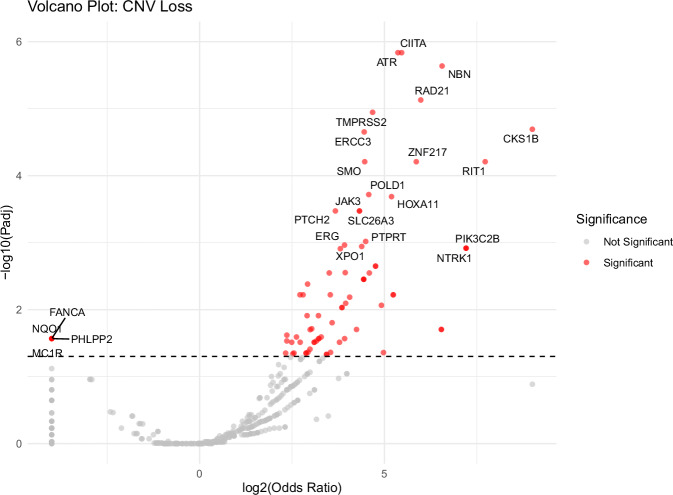
Volcano plot showing differences in copy number losses between Native American and White cohorts. The x-axis represents ${\log }_{2}$(Odds Ratio), where negative values indicate higher CNV frequencies in White samples, and positive values indicate higher frequencies in Native American samples. Red dots denote genes with significantly different CNV loss frequencies (adjusted *p*-value < 0.05).

The CNV analysis revealed population-associated differences in CNV patterns between Native American and White breast cancer patients. Figures 4 and 5 show volcano plots comparing gene-level CNV gain and loss frequencies, respectively. In both plots, the x-axis represents log_2_(odds ratio), where negative values indicate higher CNV frequencies in White patients and positive values indicate enrichment in Native American patients, and the y-axis represents the $-{\log }_{10}$ adjusted *p*-value. For visualization, log_2_(odds ratio) values corresponding to odds ratios of 0 or *∞* were truncated to the minimum and maximum x-axis limits, and adjusted *p*-values of 0 were capped at the maximum y-axis limit.

As shown in Fig. 4, 455 genes exhibited significantly more frequent CNV gains in White patients after FDR correction, whereas only 12 genes showed higher gain frequencies in Native American patients. Gain-enriched genes in White patients included *IKBKE*, which promotes NF-*κ*B and interferon signaling and supports breast cancer cell survival and immune evasion when amplified^29,30^, as well as *MDM4*, a negative regulator of the p53 pathway whose increased copy number attenuates p53-mediated tumor suppression^31,32^. In contrast, the small set of genes with higher gain frequencies in Native American patients included signaling and immune-related genes, such as *SMAD2*/*SMAD3* and *HLA-DPB1*, which are relevant to tumor progression and tumor–immune interactions^33,34^.

Compared with gains, enriched CNV losses were less numerous and showed a clearer directional tendency toward Native American patients, with 72 genes exhibiting higher loss frequencies in Native American patients compared to only 4 genes in White patients (Fig. 5). Loss-enriched genes in Native American patients primarily involved DNA damage response and genome stability, including *ATR*, a central regulator of the replication stress response^35,36^, and *RAD21*, a cohesin complex component required for DNA repair and chromosome segregation^37,38^. Genes exhibiting higher loss frequencies in White patients included *FANCA* and *NQO1*, which are involved in DNA repair and oxidative stress responses, respectively^39,40^, as well as *PHLPP2* and *MC1R*, genes with reported roles in tumor suppression and cellular signaling^41,42^.

Detailed results for gene-level CNV gains and losses are provided in Supplementary Data 2. We additionally evaluated a combined CNV change category, and the corresponding results are also summarized in Supplementary Data 2, with the volcano plot shown in Fig. S1.

Because copy number alterations frequently arise from broad chromosomal events rather than independent focal changes, statistical tests conducted at the gene level are not strictly independent. To evaluate whether the observed gene-level CNV differences were driven by large-scale chromosomal alterations, we performed an additional chromosome-arm–level CNV analysis by aggregating CNV events to chromosome-arm units in both cohorts.

At the chromosome-arm level, 32 chromosome arms exhibited significantly higher gain frequencies in White patients after FDR correction, whereas no chromosome arms reached statistical significance for losses (Tables S2–S3). For all gain-enriched chromosome arms, odds ratios were less than one, indicating a consistent enrichment of chromosomal gains in the White cohort. In contrast, although no chromosome arms reached FDR-adjusted significance for losses, the arms with the smallest adjusted *p*-values consistently showed odds ratios greater than one, suggesting a directional tendency toward higher loss frequencies in Native American patients. These arm-level patterns are concordant with the gain and loss trends observed at the gene level (Figs. 4–5).

Consistent with these arm-level findings, sample-level CNV burden analysis showed that White patients harbored substantially more chromosome-arm gains per sample than Native American patients (mean arms: 20.73 vs. 6.24; Wilcoxon *p* = 0.0014; Table S4). In contrast, Native American patients exhibited a modest but statistically significant increase in chromosome-arm loss burden compared to White patients (mean arms: 7.24 vs. 5.00; Wilcoxon *p* = 0.0339).

Together, these results indicate that the non-independence inherent to gene-level CNV tests is largely attributable to broad chromosome-arm–level gains affecting multiple genes simultaneously, particularly in White tumors. In contrast, CNV losses appear to be more focal and heterogeneous, increasing the overall loss burden in Native American patients without converging on specific chromosome arms, thereby limiting their detection in arm-level association tests.

### Mutational Signature Analysis

To analyze mutational signatures, we summarized the somatic mutation profiles into a 96-column trinucleotide count matrix and used it as input for SigProfilerExtractor^43^, which applies non-negative matrix factorization (NMF) to extract mutational signatures and their exposures across samples. The number of signatures to extract was determined by evaluating different solutions based on how closely the reconstructed profiles matched the original data (measured by average cosine distance), and how consistent the results were across repeated runs^44^. The optimal number of signatures (Fig. S2) was found to be three, which provided the best balance between low cosine distance and high stability.

**Fig. 6:**
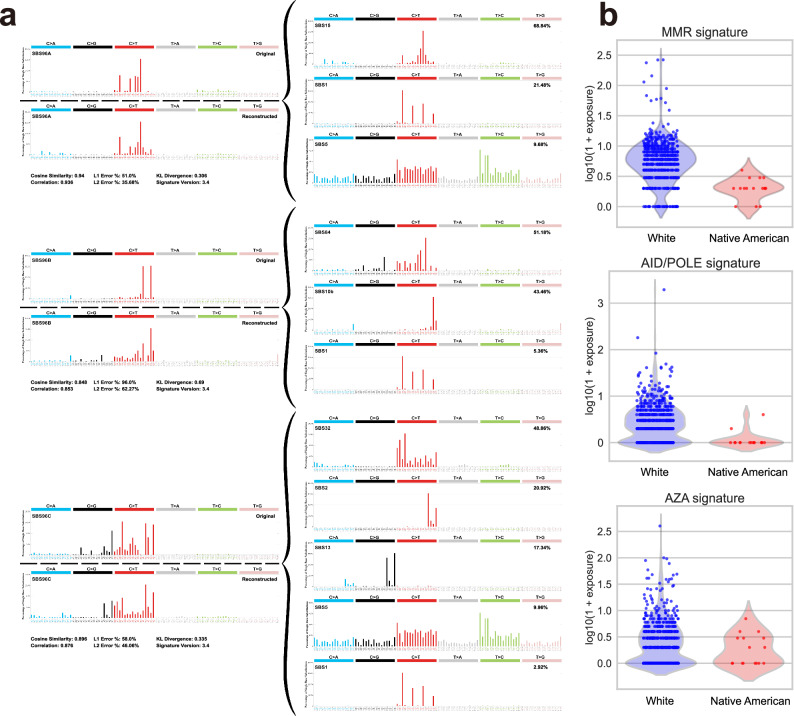
Mutational signature analysis. **a** De novo extracted mutational signatures and their COSMIC v3.4 composition. For each signature, the original and reconstructed mutation spectra are shown on the left, while the right panel presents the COSMIC signatures contributing to the composition of each extracted signature. **b** Distribution of MMR, AID/POLE, and AZA mutational signatures across Native American and White breast cancer patients. Statistical significance was assessed using the Mann-Whitney U test.

Each of the three extracted signatures mapped to known Catalog Of Somatic Mutations In Cancer (COSMIC) mutational signatures using cosine similarity analysis. To provide biologically meaningful names, we assigned each extracted signature based on the COSMIC signature with the highest relative contribution. Figure 6a illustrates the mapping.

The first extracted signature, Signature 96-A, showed the highest similarity to SBS15 (68.84%), which is linked to mismatch repair (MMR) deficiency and microsatellite instability (MSI) due to defects in *MLH1*, *MSH2*, and *MSH6*. We refer to this as the MMR signature. The second signature, Signature 96-B, was primarily driven by SBS84 (51.18%) and SBS10b (43.46%), associated with AID-mediated mutagenesis and *POLE*-driven hypermutation, respectively. Given these contributions, we name it the AID/POLE signature. The third signature, Signature 96-C, was predominantly influenced by SBS32 (48.86%), linked to azathioprine (AZA) treatment-induced C-to-T mutations. Thus, we call it the AZA signature.

To assess whether mutational signature exposures differed between the two populations, we performed the Mann-Whitney U test. Figure 6b shows the distribution of signature exposures in Native American and White breast cancer patients. The results show that exposures to the MMR and AID/POLE signatures are significantly higher in the White cohort compared to the Native American cohort, with *p*-values of 1.27 × 10^−7^ and 3.44 × 10^−5^, respectively. The elevated MMR signature suggests a higher prevalence of mismatch repair deficiency and microsatellite instability in White tumors, which may contribute to increased genomic instability and tumor immunogenicity. Similarly, higher AID/POLE exposure indicates enhanced activity of cytidine deaminases or error-prone polymerases in this group, potentially reflecting differences in mutagenic processes or environmental exposures. In contrast, exposure to the AZA signature does not differ significantly between groups (*p* = 0.23), suggesting that this mutational process may not be differentially represented between the two populations.

### Integrative Multi-Omics Pathway Analysis

Previous subsections separately examined somatic mutations, gene expression, and CNVs, where each molecular layer captures only part of the underlying biological differences. To achieve a more comprehensive understanding of population-associated molecular alterations in breast cancer, we integrated these three data types and performed an integrative multi-omics pathway analysis. In this analysis, we sought to identify pathways simultaneously enriched across somatic mutations, CNVs, and RNA expression. Such cross-layer enrichment suggests that the same biological pathway is perturbed at multiple levels and may represent a robust axis of difference between Native American and White patients.

Specifically, we evaluated KEGG pathway enrichment using genes that were differentially mutated or expressed, or that showed significantly different CNV gains or losses between Native American and White breast cancer patients. In total, we considered seven gene sets for our analysis. The mutation data included one gene set comprising 11 genes with higher mutation frequencies in Native American patients (Mutation_NA). For gene expression data, we analyzed two gene sets: 1860 DEGs upregulated in Native American patients (DEG_Up_NA) and 433 DEGs upregulated in White patients (DEG_Up_White). Genes upregulated in one population are, by definition, downregulated in the other. Therefore, differential expression in both directions is captured by the DEG_Up_NA and DEG_Up_White gene sets. The CNV data were categorized into four gene sets based on both population (Native American vs. White) and direction of CNV (gain vs. loss): 12 genes with more frequent CNV gains in Native American patients (CNV_Gain_NA), 72 genes with CNV losses in Native American patients (CNV_Loss_NA), 455 genes with CNV gains in White patients (CNV_Gain_White), and 4 genes with CNV losses in White patients (CNV_Loss_White).

**Fig. 7:**
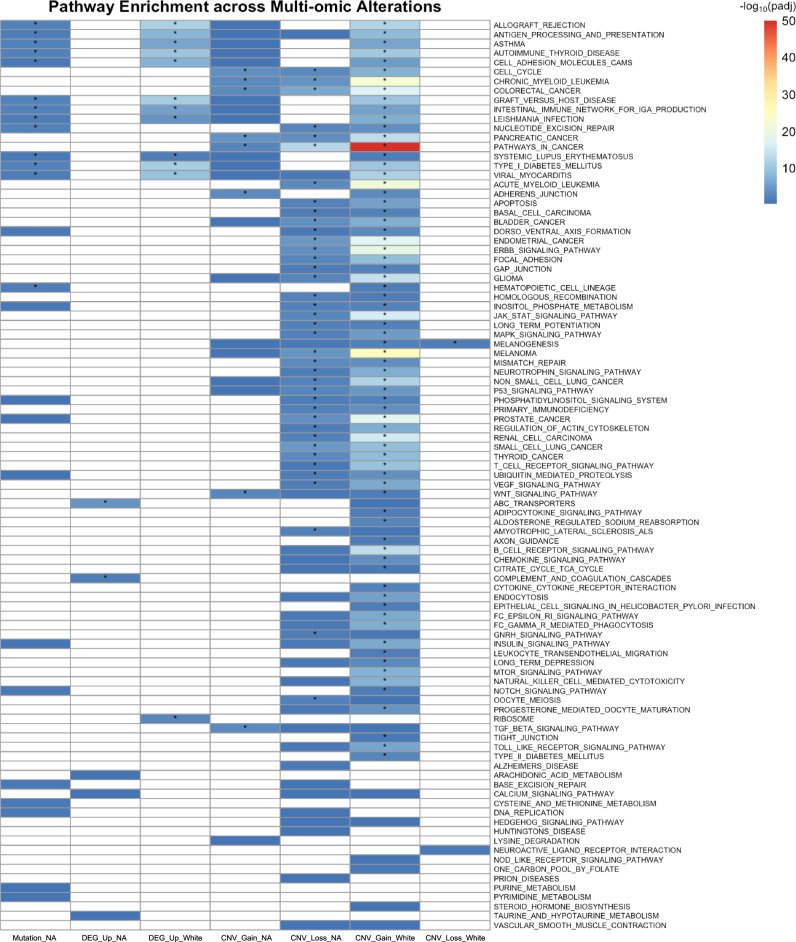
Pathway-level enrichment across multi-omic alterations (mutation, RNA expression, CNV gain/loss) between Native American and White breast cancer patients. Each row represents a KEGG pathway, and each column corresponds to one type of omic-layer alteration. Entries are colored by $-{\log }_{10}(\,{\rm{adjusted}}p{\rm{-value}})$; white entries indicate that the gene set for that omic layer does not overlap with the genes in the corresponding pathway. Genes upregulated or more frequently altered in Native American are marked with “NA,” and those enriched in White are marked with “White.” Pathways with significant enrichment are marked with a star.

Each gene set was analyzed separately for KEGG pathway enrichment, and the results were combined and shown together in Fig. 7. In this plot, each row corresponds to a KEGG pathway, and each column represents one of the seven gene sets. Color intensity reflects the enrichment significance ($-{\log }_{10}$(adjusted *p*-value)), and asterisks indicate significant results. Empty entries indicate no overlap between the gene set and the corresponding pathway. Pathways are ranked by the number of gene sets in which they are significantly enriched, from most to fewest; ties are resolved alphabetically.

To interpret Fig. 7, we first examined the top-ranked pathways (rows with the most significant enrichments), then looked vertically to determine which molecular layers (columns) contributed to each enrichment. We further referred to Supplementary Data 4 to identify the specific genes within each gene set that contributed to these pathway enrichments.

Based on this framework, we observed that several pathways exhibited coordinated enrichment across mutation, CNV, and gene expression layers (Fig. 7). Notably, some of these pathways are immune-related, such as allograft rejection, antigen processing and presentation, and graft-versus-host disease. In Native American patients, these pathways were enriched with somatic mutations in two key MHC class II genes: *HLA-DRB1* and *HLA-DRB5* (Supplementary Data 4), which are essential for presenting extracellular antigens to CD4^+^ T cells^26^. Such mutations may compromise immune recognition and facilitate tumor immune escape^45^. In contrast, tumors from White patients showed both transcriptional upregulation and CNV gains in MHC class I genes (*HLA-A* and *HLA-B*) across all three immune-related pathways. This dual-level increase may enhance antigen presentation and T cell activation, supporting stronger immune visibility and more effective immune responses^46,47^. These findings indicate that, unlike the potential immune escape suggested in Native American breast tissue samples, immune visibility may be preserved in samples from White patients, with modulation occurring at downstream signaling levels.

Another example was the CAMs pathway, which supports both the structure of epithelial tissues and communication with immune cells^48^. In Native American samples, mutations in *HLA-DRB1* and *HLA-DRB5* again suggested weakened immune recognition and impaired cell adhesion. In contrast, White samples had increased expression of *CLDN3* and *CLDN4*, which help maintain tight junctions in epithelial layers^49^. In addition, genes, such as *CD274* (PD-L1), *HLA-A*, and *HLA-B* had more frequent copy-number gains in these samples, as shown in Supplementary Data 4, pointing to enhanced regulation of both tissue structure and immune signaling^50^. These results suggest that, rather than disabling immune detection, White samples may strengthen epithelial and immune interfaces while upregulating immune checkpoints to avoid immune elimination.

We observed differences in pathways related to genomic stability. In the nucleotide excision repair pathway, Native American samples showed more mutations in *ERCC5* and *POLE*, both of which are essential for DNA damage recognition and repair^51^. These mutations may weaken the repair process and lead to more mutations accumulating in the genome^52^. In contrast, White tumors showed more CNV gains in *ERCC1* and *CUL4A*, which are involved in DNA repair through nucleotide excision and ubiquitin-mediated regulation, respectively^53,54^. These CNV gains may enhance repair capacity through gene dosage effects and structural amplification^55^. Together, these findings suggest that Native American samples may be more sensitive to common DNA-damaging chemotherapies because of impaired repair, whereas White samples may have stronger repair capacity due to higher levels of repair machinery.

## Discussion

We present, to our knowledge, the first tumor multi-omics profiling study of breast cancer in Native American women, generating matched somatic mutation, copy-number, and transcriptome data for 17 tumors. Using a population-based framework with TCGA-BRCA comparators, we delineated cross-layer differences, including higher mutation frequencies in several genes (e.g., *ARID1B*, *NOTCH4*, *HLA-DRB1*/*HLA-DRB5*) in Native American tumors, a marked enrichment of copy-number gains in White tumors, and differential expression of immune- and adhesion-related programs. Integrative analyses converged on pathways involved in antigen presentation, immune regulation, and epithelial interfaces, suggesting distinct tumor-immune ecology between groups. These findings constitute an initial reference for tumor biology in this understudied population and define testable hypotheses for larger studies.

Potential therapeutic implications should be interpreted cautiously. For example, the immune-pathway alterations (e.g., *HLA-DRB1*) could influence responses to immunotherapies, but functional validation and clinical correlation are needed^56–58^. Likewise, differences involving DNA damage-response genes (e.g., *ERCC5*) motivate evaluation of DNA repair-targeted strategies and response to DNA-damaging agents, which require prospective outcome data^59,60^.

This work has limitations. The cohort is modest (17 Native American tumors), limiting statistical power and generalizability across diverse Native communities. Cross-cohort platform differences (panel-based DNA with panel-derived copy-number calls in this study vs. TCGA whole-exome/SNP-array CNV; RNA-seq in both) may contribute residual batch effects despite harmonization (restriction to the 648 panel genes for mutation/CNV analyses and rank-based normalization for RNA-seq). Mutational signatures were inferred from a targeted 648-gene panel; panel-based catalogs contain fewer mutations and nonuniform trinucleotide contexts relative to WES/WGS, which can reduce signature stability and bias exposure estimates. Accordingly, group comparisons of signature exposures should be viewed as exploratory, even with FDR adjustment. Moreover, we cannot rule out the influence of societal and/or environmental influences, in addition to ancestry, that may be contributors to the unique somatic biology observed in this Native American population. Additionally, clinical covariates (e.g., intrinsic subtype, disease stage, treatment history, and comorbidities) were unavailable or incompletely captured across the Native American samples, as clinical information was largely limited to pathology reports, precluding adjustment for potential confounding. Finally, ancestry in this study was self-reported, and genetic ancestry could not be directly assessed because paired germline blood samples were unavailable. Future work should incorporate genetic ancestry inference and prospective accrual with standardized assays and outcome data.

Our study has several notable strengths. First, it focuses on a historically understudied population. This study generates matched somatic mutation, copy-number, and transcriptomic profiles from breast tumors in Native American women. In doing so, it addresses a major gap in cancer genomics resources that are dominated by European-ancestry cohorts and include vanishingly few Native American cases. Second, the study adopts an integrative multi-omics framework that enables the identification of population-associated differences supported across multiple molecular layers, rather than relying on a single data modality. The convergence of signals across DNA mutation, CNV, RNA expression, and pathway-level analyses enhances biological interpretability and confidence in the findings, even in the context of a modest cohort size. Third, the analytical strategy is explicitly designed to be robust given the constraints of sample size and platform heterogeneity. We employed standard, well-established statistical tests with FDR control, together with rank-based expression analyses to mitigate cross-platform effects, and complemented primary findings with multiple sensitivity and robustness analyses. These choices prioritize reproducibility and interpretability while avoiding overstatement of effect sizes or causal inference. As such, the study is positioned as an initial molecular reference that yields reproducible population-associated signals and motivates larger, harmonized follow-up studies.

To further evaluate the robustness of our findings under the limitations of cohort size, platform heterogeneity, and statistical assumptions, we conducted a series of complementary sensitivity and validation analyses across the DNA mutation, CNV, and RNA expression layers. For the DNA mutation analysis, we performed a non-parametric bootstrap analysis on genes identified as significant in the primary Fisher’s exact tests. Across 5000 bootstrap resamples within each population, the estimated log odds ratios remained highly stable, with nearly perfect directional consistency for all significant genes (Fig. S3, Table S1), indicating that the observed mutation enrichments are robust to sampling variability. For the CNV analysis, we explicitly addressed the non-independence of gene-level tests by incorporating a chromosome-arm–level analysis in the main Results section. The arm-level results revealed that widespread gene-level CNV enrichments are largely driven by broad chromosomal gain events, particularly in White tumors, while loss events in Native American samples appear more heterogeneous and less convergent at the arm level. These findings provide a mechanistic explanation for the gene-level CNV results and confirm that they are not artifacts of multiple correlated tests. For RNA expression, we conducted an independent robustness analysis using the Rank-In framework^61^, which mitigates cross-platform and normalization effects by operating on within-sample adjusted expression ranks. As shown in Fig. S4, the vast majority of differentially expressed genes identified in the primary analysis were independently validated by Rank-In, with highly concordant effect directions, demonstrating that the reported expression differences are robust to the choice of expression scale and effect size definition. More methodological details and results regarding these analyses are provided in the Supplementary Material. Taken together, these sensitivity analyses provide convergent evidence that the key DNA mutation, CNV, and RNA expression differences reported in this study are stable and reproducible across multiple analytical perturbations, supporting the reliability of the integrative conclusions despite the modest size of the Native American cohort.

In sum, our dataset and analyses supply an initial multi-omics resource for Native American breast tumors, highlight reproducible molecular signals across layers, and motivate community-engaged, harmonized studies with larger cohorts and outcome data to determine prognostic and therapeutic relevance.

## Methods

### Study Design and Data Collection

This study investigates genomic and transcriptomic differences in breast cancer between Native American and White populations by integrating data from somatic mutations, CNVs, and RNA-seq. We analyzed tumor samples from Native American patients and compared them to publicly available White breast cancer data from TCGA.

The study was conducted in accordance with the Declaration of Helsinki and the U.S. Common Rule. The protocol was approved by the University of Notre Dame Institutional Review Board (University of Notre Dame Institutional Review Board; IRB# 20-02-5847). Archival breast tumor tissues were identified at MultiCare Health System and transferred under an IRB Authorization Agreement. Participants provided informed consent for retrospective research use of their tissue; all pathology reports and tissue specimens were de-identified prior to transfer to the study investigators. In working with Native American cohorts, we followed institutional guidance on Indigenous data governance; individual-level genomic data are not shared.

This retrospective analysis of formalin-fixed paraffin-embedded (FFPE) tissue blocks used archival tissues obtained from the MultiCare Health System pathology group from cases examined between 2012 and 2014. The MultiCare electronic medical record was queried using the Epic Slicer Dicer tool for race/ethnicity data to retrospectively identify breast cancer patients who self-identified as belonging to Native communities (American Indian and/or Alaska Native). A total of 17 patients for whom archived tumor tissue was available, and consent was obtained, were included in the study.

The pathology reports and FFPE blocks were de-identified prior to shipment to the study investigators at the University of Notre Dame. Because this was a retrospective, archival study, complete information on the total number of potentially eligible Native American breast cancer cases during this period was not available. No additional clinical or demographic data were available beyond pathology reports.

Tissue blocks from Native American breast cancer patients were analyzed using Tempus sequencing assays. Targeted DNA sequencing and copy number alteration (CNA) detection were performed using the clinically validated Tempus xT v4 next-generation sequencing (NGS) assay, which targets 648 cancer-related genes and is designed to detect single-nucleotide variants (SNVs), insertions and deletions (indels), copy number alterations, and gene rearrangements^62,63^. The full list of the 648 genes included in the panel is provided in the Tempus xT Validation Document.

DNA sequencing was then performed on the Illumina NovaSeq 6000 platform, generating paired-end 2 × 126 bp reads with an average sequencing depth of approximately 500 × for tumor samples and 150 × for matched normal samples. Variant calling and quality control were conducted by the sequencing provider as part of the standard Tempus xT v4 clinical pipeline using validated tools, including FreeBayes^64^ and Pindel^65^. For downstream analyses, we used the provider-released somatic variants identified by FreeBayes and did not apply additional filtering based on variant allele frequency (VAF) thresholds or tumor purity adjustments. Copy number alterations were identified using the Tempus xT v4 pipeline, which provides segment-level CNA annotations.

RNA sequencing was performed using the Tempus xR whole-transcriptome sequencing (WTS) assay. Paired-end 2 × 75 bp sequencing was conducted, generating approximately 50 million reads per sample. Reads were aligned to the GRCh37 (hg19) reference genome by the sequencing provider. Following alignment and internal processing, gene-level RNA-seq expression quantification, including both raw and normalized expression values, was generated and provided for downstream analyses. No additional RNA-seq re-quantification was performed in this study.

For comparison, genomic and transcriptomic data from breast cancer patients categorized as White in TCGA were obtained via the Genomic Data Commons (GDC) Data Portal^66^. The downloaded dataset included 689 samples with somatic mutation data, 727 samples with CNV data, and 754 samples with RNA-seq data. Based on TCGA self-reported ethnicity annotations, Hispanic or Latino participants were included across the datasets, with 28 in the somatic mutation dataset, 32 in the CNV dataset, and 34 in the RNA-seq dataset.

### DNA Mutation Analysis

Somatic mutation data for the Native American cohort were preprocessed using the Variant Effect Predictor (VEP)^67^ for functional annotation, including allele frequencies (AF) and mutation consequences. The resulting VCF files were converted to Mutation Annotation Format (MAF) using vcf2maf^68^ to match the format of the White cohort data and ensure consistency in downstream analyses.

To improve data quality, we applied several filtering steps. First, we removed variants that are common in the general population by excluding those with an AF greater than 0.0005 in any subpopulation of the Genome Aggregation Database (gnomAD)^69^. We then kept only protein-altering somatic mutations, such as missense, nonsense, frameshift, in-frame insertions or deletions, splice site changes, and changes affecting the start of translation. Mutations that do not change the protein (silent mutations) or are located in non-coding regions (such as 3’UTR, 5’UTR, introns, and nearby flanking regions) were excluded. Additionally, only mutations within the 648-gene panel were retained to ensure comparability between cohorts.

Fisher’s exact test was used to assess the significance of differences in mutation frequency at the individual gene level between the Native American and TCGA White cohorts. The resulting *p*-values were adjusted using the BH method to control FDR. Genes with adjusted *p*-values < 0.1 were considered to have significantly different mutation frequencies between the two populations. In addition to statistical testing, an oncoplot was used to visualize the distribution and frequency of somatic mutations across samples, highlighting the most frequently mutated genes and their mutation types.

### CNV Analysis

Gene-level CNV annotations were obtained by assigning gain, loss, or neutral CNV segments to genes based on their genomic coordinates using the TxDb.Hsapiens.UCSC.hg19.knownGene database^70^. If a gene in a given sample overlapped with multiple CNV segments with conflicting statuses (e.g., both gain and loss), its CNV status was considered invalid and excluded. Only genes from the 648-gene panel with a valid CNV status were retained for downstream analysis.

For each gene, the number of samples with valid CNV status (gain, loss, or neutral) was counted separately for the Native American and White cohorts, and these counts were used to calculate CNV frequencies within each group. Gain frequency was defined as the proportion of samples with a gain call among all samples with valid CNV status for that gene; loss frequency was calculated similarly. We also defined a combined “CNV Change” category, including any sample annotated as either gain or loss. Its frequency was calculated as the proportion of CNV Change samples among all valid samples for that gene.

The frequencies of CNV events (gain, loss, and CNV Change) were compared between Native American and White populations using Fisher’s exact test. Contingency tables were constructed for each gene, and separate tests were performed for each CNV event type. Adjusted *p*-values were obtained using the BH procedure to control the FDR, with adjusted *p*-values less than 0.05 considered statistically significant. For genes with significant differences, the population with the higher frequency of CNV events was identified.

In the chromosome-arm–level CNV analysis, CNV calls from the Native American cohort (available at the segment level) and from the White cohort (available at the gene level) were independently collapsed to chromosome-arm units based on genomic coordinates, ensuring comparability across platforms. For each sample, an arm-level gain or loss was defined if at least one panel gene on the corresponding chromosome arm exhibited the respective CNV event. Arm-level gain and loss frequencies were compared between Native American and White cohorts using Fisher’s exact tests with FDR correction. In addition, sample-level CNV burden was quantified as the number of chromosome arms affected by gains or losses per sample and compared between groups using Wilcoxon rank-sum tests.

### RNA Sequencing Analysis

We received normalized RNA-seq expression matrices and converted the Ensembl gene IDs to HGNC gene symbols using the EnsDb.Hsapiens.v75 database^71^. Genes with low average expression (mean normalized value < 10) across both Native American and White cohorts were excluded from further analysis. Expression values within each sample were then rank-transformed. Specifically, gene expression values were replaced by their ranks based on expression levels within the same sample, with tied values assigned the average rank. Since the two cohorts were sequenced on different platforms, this transformation helped address potential platform-specific variation by focusing on relative rather than absolute expression levels.

Differential expression analysis between the Native American and White populations was conducted using the Mann-Whitney U test to compare the distributions of gene expression ranks. For each gene, raw *p*-values from the test were corrected for multiple hypothesis testing using the BH procedure to control the FDR. Genes with an adjusted *p*-value less than 0.01 and a log2 fold change (computed on the original normalized expression values prior to rank transformation; absolute value) of at least 2 were considered statistically significant and identified as differentially expressed genes (DEGs).

Gene Ontology (GO) enrichment analysis of DEGs focused on the Biological Process (BP) category. Additionally, Kyoto Encyclopedia of Genes and Genomes (KEGG) pathway analysis used gene sets obtained from the Molecular Signatures Database (MSigDB)^72^. Both analyses were implemented using the clusterProfiler R package^73^, with pathways considered significantly enriched at an FDR-adjusted *p*-value less than 0.05.

### Mutational Signature Analysis

Single-base substitution (SBS) mutational signatures were extracted using SigProfilerExtractor^43^ in Python, based on the GRCh37 reference genome. The analysis considered six types of SBS events: C>T, C>A, C>G, T>C, T>A, and T>G^74^. For each mutation, the bases immediately preceding and following the mutated site were included to define its trinucleotide context. This resulted in 96 distinct trinucleotide mutation types, combining the six substitution classes with all possible flanking base combinations. We counted how many mutations fell into each of these 96 types across all Native American and White samples and organized the results into a matrix with 96 columns-one for each mutation type. This matrix was then used as input for SigProfilerExtractor to extract the mutational signatures.

SigProfilerExtractor applied non-negative matrix factorization (NMF) to decompose the mutational catalog into candidate mutational signatures and their corresponding exposures across samples^75^. The number of extracted signatures ranged from 1 to 10. For each solution, the tool performed multiple rounds of resampling and reconstruction to assess stability, reproducibility, and goodness-of-fit. The final number of signatures was automatically selected based on internal metrics, including signature stability, reconstruction error, and Frobenius norm^44^.

Extracted signatures were then compared to known SBS signatures from the Catalog of Somatic Mutations in Cancer (COSMIC, version 3.4)^76^ using cosine similarity. Each signature was matched to the most similar COSMIC reference and renamed accordingly.

To assess whether mutational signature exposures differed between Native American and White breast cancer patients, we used the Mann-Whitney U test. For each signature, we compared its exposure values between the two groups. The test was performed in Python using the mannwhitneyu function from the scipy library, with a two-sided test and a significance level of *p* < 0.05.

### Computational Tools

All computational analyses were conducted using R (v4.4.3) and Python (v3.10.13). Key packages included TCGAbiolinks for data retrieval^77^, VEP for variant annotation^67^, clusterProfiler for functional enrichment^73^, and SigProfilerExtractor for mutational signature analysis with COSMIC v3.4^43^. maftools^68^ was used for somatic mutation visualization, including oncoplot for mutation frequency comparisons. Data visualization was also carried out using ggplot2^78^.

## Supplementary information

Supplementary information

Supplementary Data 1

Supplementary Data 2

Supplementary Data 3

Supplementary Data 4

## Data Availability

All processed data supporting the findings of this study are publicly available on Zenodo (10.5281/zenodo.17109463). The dataset includes somatic mutation results (Supplementary Data 1), CNV results (Supplementary Data 2), differential expression results (Supplementary Data 3), integrated KEGG pathway enrichment results (Supplementary Data 4), and aggregated average gene expression values for Native American breast cancer samples. All shared materials are aggregated summary-level data that do not contain any personally identifiable information. This approach respects Native American data sovereignty and participant privacy, while enabling verification and reproducibility of our findings. TCGA data used for comparison were obtained from the NCI Genomic Data Commons (GDC) Data Portal (project TCGA-BRCA), and all analyses were performed using open-access files.

## Code availability

All analysis code (R and Python) and figure-generation scripts are hosted at our GitHub repository (https://github.com/Fangfang-Guo/BRCA_NA). The repository is publicly accessible.
