# Supplementary material for: Molecular profiling of breast cancer in native American women reveals distinct genomic and transcriptomic features
Source: NPJ Precis Oncol. 2026 Mar 17;10:175. doi: 10.1038/s41698-026-01373-6 (PMC13144316; doi:10.1038/s41698-026-01373-6)
Supplement: Supplementary file 1 — Supplementary information [file 41698_2026_1373_MOESM1_ESM.pdf]

Supplementary Material

Supplementary Analysis

Bootstrap robustness analysis for DNA mutation effect sizes

To assess the robustness of gene-level mutation effect size estimates under the observed cohort sizes, we performed a non-parametric bootstrap analysis focusing on the log odds ratios (logOR) obtained from the Fisher's exact test in the DNA mutation analysis, specifically for genes identified as significant in the primary analysis.

Native American and Caucasian samples were resampled with replacement within each population, preserving the original cohort sizes. For each bootstrap replicate, gene-level mutation frequencies were recalculated and a log odds ratio was recomputed using the same contingency-table framework as in the primary analysis. This procedure was repeated 5,000 times.

For each gene, the bootstrap distribution of logOR values was summarized by its median and a 95% percentile-based confidence interval. In addition, directional consistency was quantified as the proportion of bootstrap replicates in which the sign of the logOR matched that of the original estimate. Genes with high directional consistency indicate stable effect directions despite resampling variability.

The bootstrap analysis demonstrates strong stability of the estimated odds ratios. As summarized in Table S1, all genes identified as significant in the primary analysis exhibit highly consistent effect directions across resamples, with directional consistency equal to 1.000 for 10 of 11 genes and remaining above 0.997 for *ARID1B*. In addition, the median bootstrap

logOR closely matches the observed logOR for all genes, and the percentile-based 95% confidence intervals do not include zero, corroborating the significance of the mutation-rate differences identified in the primary analysis.

Consistent with these summary statistics, the bootstrap distributions for representative genes shown in Figure S3 are approximately bell-shaped and centered around the observed logOR values, with symmetric tails and no evidence of sign instability under resampling. Taken together, the quantitative summaries in Table S1 and the bootstrap distributions shown in Figure S3 provide evidence that the reported mutation enrichments are robust to sampling variability.

Rank-In robustness analysis for RNA differential expression

When RNA expression profiles are generated using different processing pipelines or platforms, effect sizes computed directly from normalized expression values (for example, $\log_2$ fold change) may partially reflect technical variation rather than true cohort-level biological differences. To evaluate the robustness of our differential expression results to this potential source of bias, we performed an additional analysis using the Rank-In framework [**?**], which is specifically designed to mitigate cross-platform and cross-processing effects by operating on within-sample adjusted expression ranks instead of normalized expression values.

The adjusted rank expression matrix was generated using the official Rank-In executable provided by the authors (`http://www.badd-cao.net/rank-in/index.html`). Differential expression analysis was then conducted following the Rank-In methodology. For each gene, a Wilcoxon rank-sum test was applied to compare adjusted ranks between Native American and White samples, and p-values were adjusted using the Benjamini–Hochberg procedure to control the false discovery rate (FDR < 0.01).

We compared the differentially expressed genes (DEGs) identified by our original analysis (FDR < 0.01 and $|\log_2 \mathrm{FC}| \geq 2$) with those identified by the Rank-In analysis. As shown in Figure S4, the vast majority of DEGs detected by the original method are also supported by Rank-In. Specifically, among all original-method DEGs, 2214 out of 2293 genes (96.6%) are also significant under Rank-In. When stratified by direction, 1784 of 1860 Native American upregulated genes (95.9%) and 428 of 433 White upregulated genes (98.8%) are validated by Rank-In.

We further examined concordance in effect direction. In the Rank-In framework, the sign of the difference in mean adjusted ranks between groups (DeltaRank) is used to indicate differential expression direction. Comparison with the sign of the $\log_2$ fold change from our original analysis showed very high directional consistency: among the 2,214 overlapping DEGs, 2,212 genes (99.9%) exhibited concordant directions, with only two genes showing discordant signs (Figure S4). This high directional agreement indicates that the large-magnitude $\log_2$ fold changes identified using normalized expression values are unlikely to be driven primarily by platform- or processing-specific effects.

The Rank-In analysis identifies a larger number of DEGs overall, as expected given that it does not impose an explicit effect size threshold. In contrast, our original analysis intentionally applied a stringent $|\log_2 \mathrm{FC}| \geq 2$ criterion to focus on genes with large-magnitude expression differences that are more likely to be biologically meaningful and robust across platforms. As a result, the original DEG set represents a stringently filtered, high-effect-size subset of the Rank-In results.

Supplementary Data

Supplementary Data 1

File `Supplementary_Data_1.csv`. Summary of significant gene mutations (Padj < 0.1) identified by Fisher's exact test comparing Native American and White samples. The file reports the number of mutated and non-mutated individuals in each population, along with the associated p-values and adjusted p-values.

Supplementary Data 2

File `Supplementary_Data_2.xlsx`. Detailed results of the copy number variation (CNV) analysis comparing Native American and White breast cancer patients. The following three tabs report statistically significant CNV events:

- `Gain`: Genes with significantly higher CNV gain frequencies in one group compared to the other.
- `Loss`: Genes with significantly higher CNV loss frequencies in one group.
- `Change`: Genes with significant differences in CNV gain/loss status between Native American and White patients.

All results are based on Fisher's exact test. Each table includes gene symbols, odds ratios, p-values, adjusted p-values, and the population (Native American or White) in which CNV gain or loss is more frequent.

Supplementary Data 3

File `Supplementary_Data_3.csv`. Differentially expressed genes between Native American and White breast cancer patients. The file lists all genes with an absolute $\log_2$ fold change ≥ 2 and an adjusted p-value < 0.01. The table includes gene symbols, $\log_2$ fold changes, raw p-value, adjusted p-value, and the population (Native American or White) in which the gene is upregulated.

Supplementary Data 4

File Supplementary_Data_4.csv. This table provides gene-level annotations to complement the pathway-level visualization in Figure 7 of the main text. Each row corresponds to a KEGG pathway, and each column represents one of the seven input gene sets: Mutation_NA, DEG_Up_NA, DEG_Up_White, CNV_Gain_NA, CNV_Loss_NA, CNV_Gain_White, and CNV_Loss_White. The entries in each cell list the genes from the respective gene set that overlap with the given pathway, only if the enrichment was statistically significant (adjusted $p < 0.05$) in that context.

Supplementary Tables

Table S1: Bootstrap robustness analysis of mutation odds ratios. For each gene significant in the primary analysis (FDR < 0.1), the table reports the observed log odds ratio (logOR), the median bootstrap logOR, percentile-based 95% confidence intervals, and the proportion of bootstrap resamples with the same effect direction as the observed estimate.

Gene	logOR	Boot. median	$CI_{2.5\%}$	$CI_{97.5\%}$	Dir. consistency
BCOR	3.089	3.080	1.100	4.456	1.0000
DNMT3A	3.234	3.219	1.269	4.708	1.0000
ERCC5	5.405	5.405	3.674	6.408	1.0000
FANCL	4.305	4.305	2.061	6.131	1.0000
FOXO1	4.708	4.796	2.627	6.408	1.0000
HLA-DRB1	6.408	6.408	5.405	7.340	1.0000
HLA-DRB5	5.808	5.808	3.674	6.659	1.0000
INPP4B	3.605	3.605	1.723	5.308	1.0000
NOTCH4	3.563	3.563	2.253	4.849	1.0000
POLE	3.234	3.234	1.269	4.690	1.0000
ARID1B	2.550	2.550	1.140	3.599	0.9972

Table S2: Chromosome-arm–level CNV gains (NA vs. White), sorted by FDR (OR < 1 indicates enrichment in White; horizontal line denotes FDR $= 0.05$).

Chromosome arm	NA (gain)	White (gain)	OR	FDR
chr8p	0 / 17	418 / 727	0.000	0.000
chr5p	0 / 17	403 / 727	0.000	0.000
chr11p	0 / 17	359 / 727	0.000	0.000
chr2q	0 / 17	356 / 727	0.000	0.000
chr4q	0 / 17	351 / 727	0.000	0.000
chr3q	1 / 17	406 / 727	0.050	0.000
chr11q	2 / 17	445 / 727	0.085	0.000
chr3p	0 / 17	332 / 727	0.000	0.000
chr9p	0 / 17	329 / 727	0.000	0.000
chr7q	1 / 17	395 / 727	0.053	0.000
chr12q	1 / 17	383 / 727	0.056	0.000
chr5q	1 / 17	374 / 727	0.059	0.000
chr20p	1 / 17	364 / 727	0.062	0.001
chr7p	2 / 17	408 / 727	0.105	0.001
chr21q	1 / 17	356 / 727	0.065	0.001
chr14q	1 / 17	354 / 727	0.066	0.001
chr10q	1 / 17	353 / 727	0.066	0.001
chr2p	1 / 17	353 / 727	0.066	0.001
chr4p	0 / 17	278 / 727	0.000	0.001
chr8q	5 / 17	518 / 727	0.169	0.001
chr6q	1 / 17	324 / 727	0.078	0.002
chr18p	0 / 17	263 / 727	0.000	0.002
chr18q	1 / 17	308 / 727	0.085	0.004
chr10p	2 / 17	364 / 727	0.133	0.004
chr19q	3 / 17	399 / 727	0.177	0.005
chr15q	2 / 17	334 / 727	0.157	0.010
chr9q	2 / 17	330 / 727	0.161	0.010
chr20q	5 / 17	452 / 727	0.254	0.017
chrXp	2 / 17	310 / 727	0.180	0.019
chr13q	2 / 17	308 / 727	0.182	0.019
chr12p	4 / 17	369 / 727	0.299	0.044
chr16p	7 / 17	503 / 727	0.312	0.046
chr1q	11 / 17	618 / 727	0.324	0.050
chr6p	5 / 17	388 / 727	0.365	0.116
chr17p	2 / 17	231 / 727	0.287	0.147
chr16q	2 / 17	233 / 727	0.283	0.147
chr17q	7 / 17	446 / 727	0.442	0.168
chrXq	4 / 17	319 / 727	0.394	0.172
chr1p	11 / 17	387 / 727	1.610	0.569
chr13p	0 / 17	0 / 727	0.000	1.000
chr14p	0 / 17	0 / 727	0.000	1.000
chr15p	0 / 17	0 / 727	0.000	1.000
chr21p	0 / 17	0 / 727	0.000	1.000
chr22p	0 / 17	0 / 727	0.000	1.000
chr19p	9 / 17	372 / 727	1.074	1.000
chr22q	6 / 17	275 / 727	0.897	1.000

Table S3: Chromosome-arm–level CNV losses (NA vs. White), sorted by FDR (OR < 1 indicates enrichment in White; no chromosome arms are significant after FDR correction.

Chromosome arm	NA (loss)	White (loss)	OR	FDR
chr7q	5 / 17	53 / 727	5.276	0.147
chr2p	4 / 17	33 / 727	6.435	0.147
chr2q	6 / 17	83 / 727	4.219	0.147
chr3q	4 / 17	38 / 727	5.553	0.147
chr9q	5 / 17	76 / 727	3.560	0.280
chr19q	3 / 17	40 / 727	3.669	0.398
chr8q	3 / 17	40 / 727	3.669	0.398
chr8p	9 / 17	230 / 727	2.428	0.398
chr6q	7 / 17	159 / 727	2.497	0.398
chr17p	7 / 17	193 / 727	1.935	0.786
chr12p	3 / 17	63 / 727	2.255	0.786
chr1p	1 / 17	139 / 727	0.265	0.786
chr22q	1 / 17	153 / 727	0.235	0.786
chr13q	6 / 17	157 / 727	1.978	0.786
chr4p	4 / 17	94 / 727	2.069	0.786
chr9p	4 / 17	99 / 727	1.950	0.786
chr4q	4 / 17	103 / 727	1.862	0.786
chr10q	4 / 17	105 / 727	1.821	0.786
chr17q	4 / 17	110 / 727	1.724	0.790
chr11p	3 / 17	79 / 727	1.756	0.985
chr7p	1 / 17	23 / 727	1.911	0.985
chr14q	3 / 17	95 / 727	1.425	1.000
chr5q	1 / 17	100 / 727	0.392	1.000
chr16p	1 / 17	31 / 727	1.402	1.000
chr11q	5 / 17	168 / 727	1.386	1.000
chr21q	1 / 17	39 / 727	1.102	1.000
chr6p	2 / 17	67 / 727	1.313	1.000
chr19p	2 / 17	70 / 727	1.251	1.000
chr12q	2 / 17	71 / 727	1.232	1.000
chrXp	1 / 17	95 / 727	0.416	1.000
chr15q	3 / 17	101 / 727	1.328	1.000
chr3p	3 / 17	106 / 727	1.255	1.000
chrXq	3 / 17	108 / 727	1.228	1.000
chr18p	1 / 17	53 / 727	0.795	1.000
chr10p	0 / 17	18 / 727	0.000	1.000
chr18q	1 / 17	80 / 727	0.506	1.000
chr20q	0 / 17	28 / 727	0.000	1.000
chr13p	0 / 17	0 / 727	0.000	1.000
chr14p	0 / 17	0 / 727	0.000	1.000
chr15p	0 / 17	0 / 727	0.000	1.000
chr21p	0 / 17	0 / 727	0.000	1.000
chr22p	0 / 17	0 / 727	0.000	1.000
chr1q	0 / 17	16 / 727	0.000	1.000
chr20p	0 / 17	25 / 727	0.000	1.000
chr5p	0 / 17	29 / 727	0.000	1.000
chr16q	6 / 17	264 / 727	0.957	1.000

Table S4: Comparison of sample-level chromosome-arm CNV burden between Native American (NA) and White samples. CNV burden is defined as the number of chromosome arms affected by gains or losses per sample.

CNV type	Group	Median arms	Mean arms	Wilcoxon p
Gain	NA	5.0	6.24	0.0014
	White	19.0	20.73	
Loss	NA	6.0	7.24	0.0339
	White	3.0	5.00	

Supplementary Figures

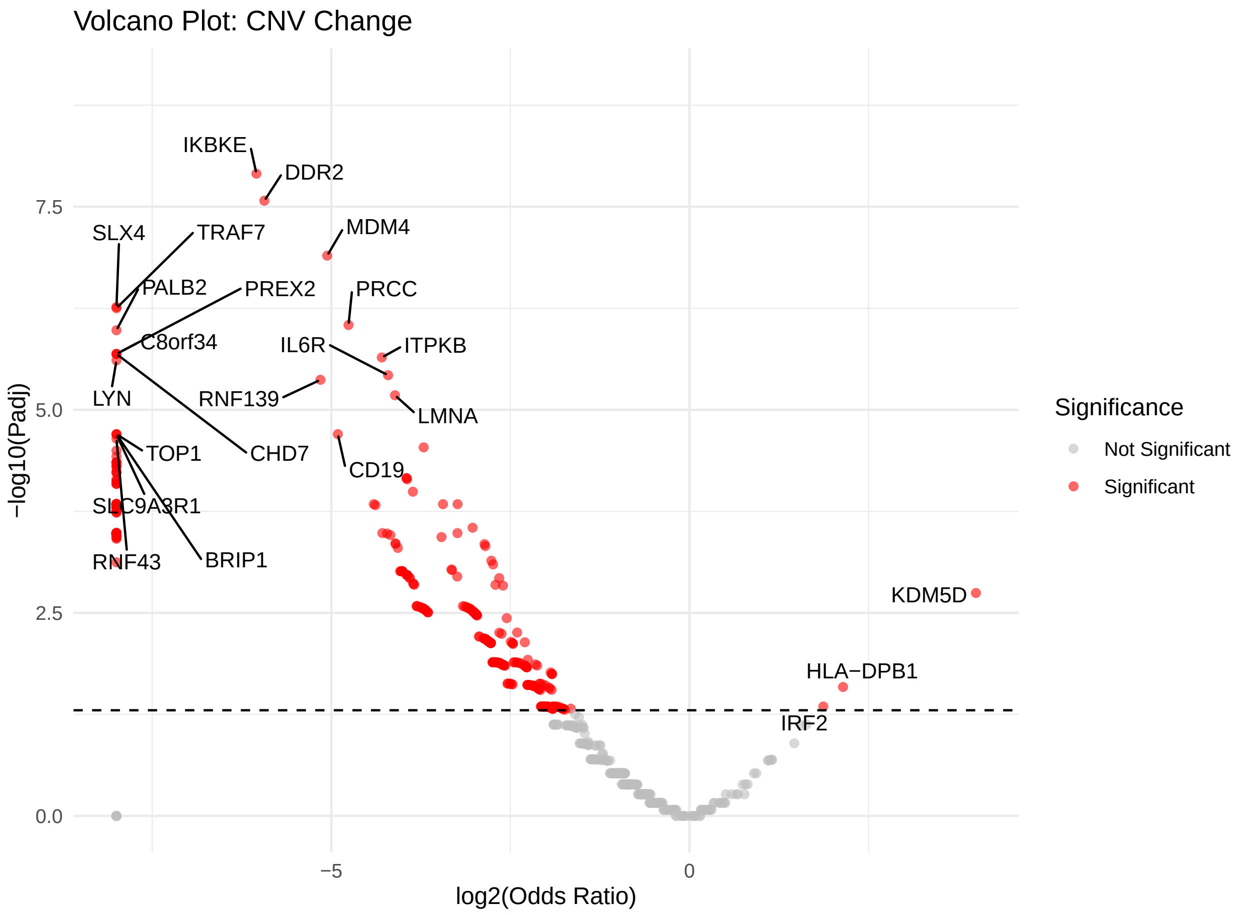

Figure S1: Volcano plot illustrating differences in CNVs between Native American and White cohorts. The x-axis represents $\log_2$(odds ratio), where negative values indicate higher CNV frequencies in White samples and positive values indicate higher frequencies in Native American samples. Red dots denote genes with significantly different CNV frequencies (adjusted p-value < 0.05). Genes with odds ratios of 0 or ∞ are plotted at the respective bounds of the x-axis, and adjusted p-values of 0 are capped at the top of the y-axis.

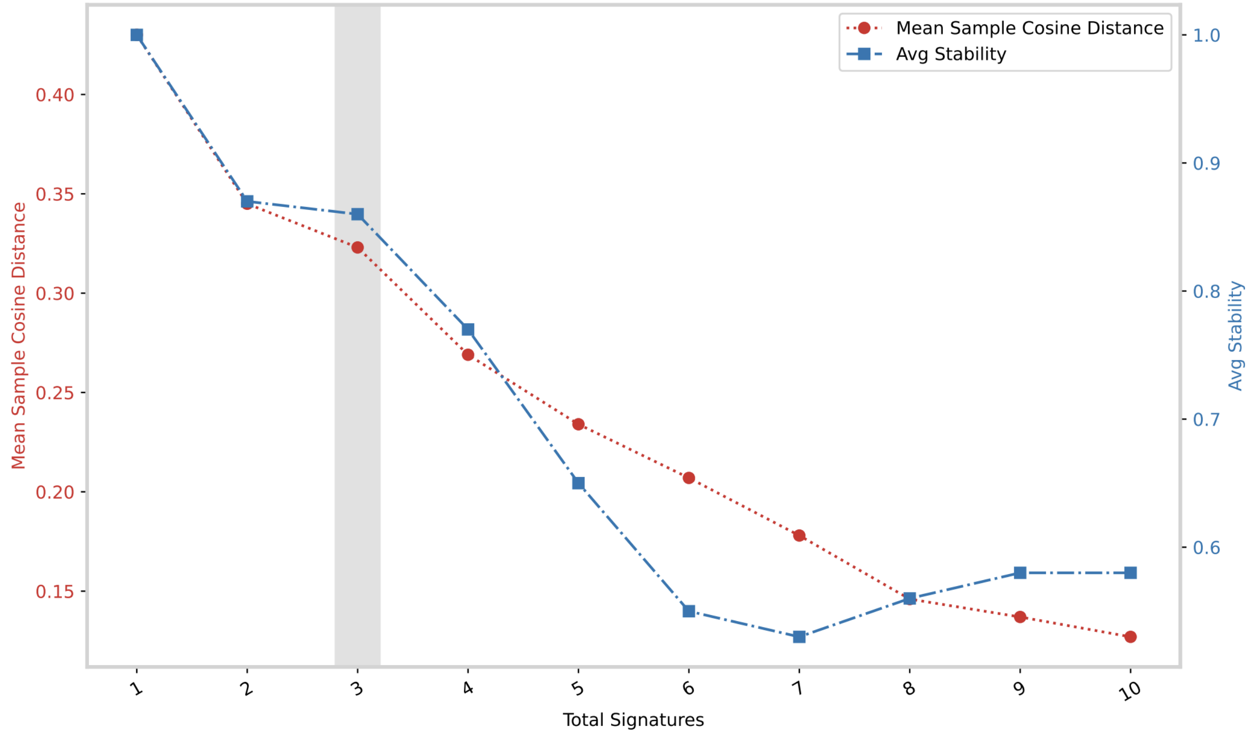

Figure S2: Selection of the optimal number of mutational signatures based on mean sample cosine distance (red) and average stability (blue). The shaded region indicates the selected solution with three signatures.

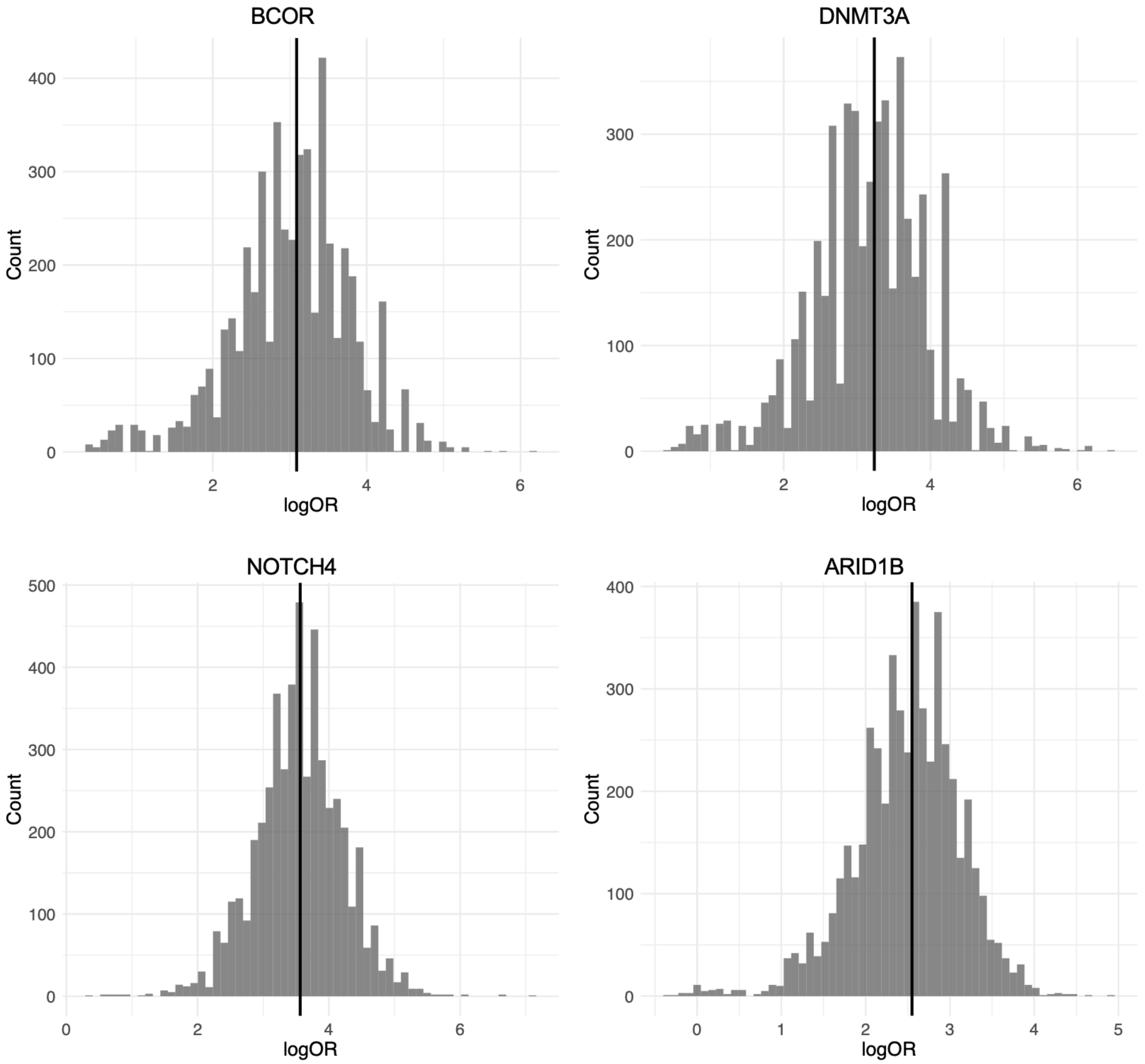

Figure S3: Bootstrap distributions of log odds ratios (logOR) for representative genes identified in the DNA mutation analysis. For each gene, histograms show the distribution of NA–White logOR values obtained from 5,000 bootstrap resamples of the Native American and White cohorts. The vertical black line indicates the observed logOR from the primary analysis. Across all shown genes, the bootstrap distributions are centered near the observed estimate and exhibit highly consistent effect direction, supporting the robustness of the inferred mutation enrichments under resampling.

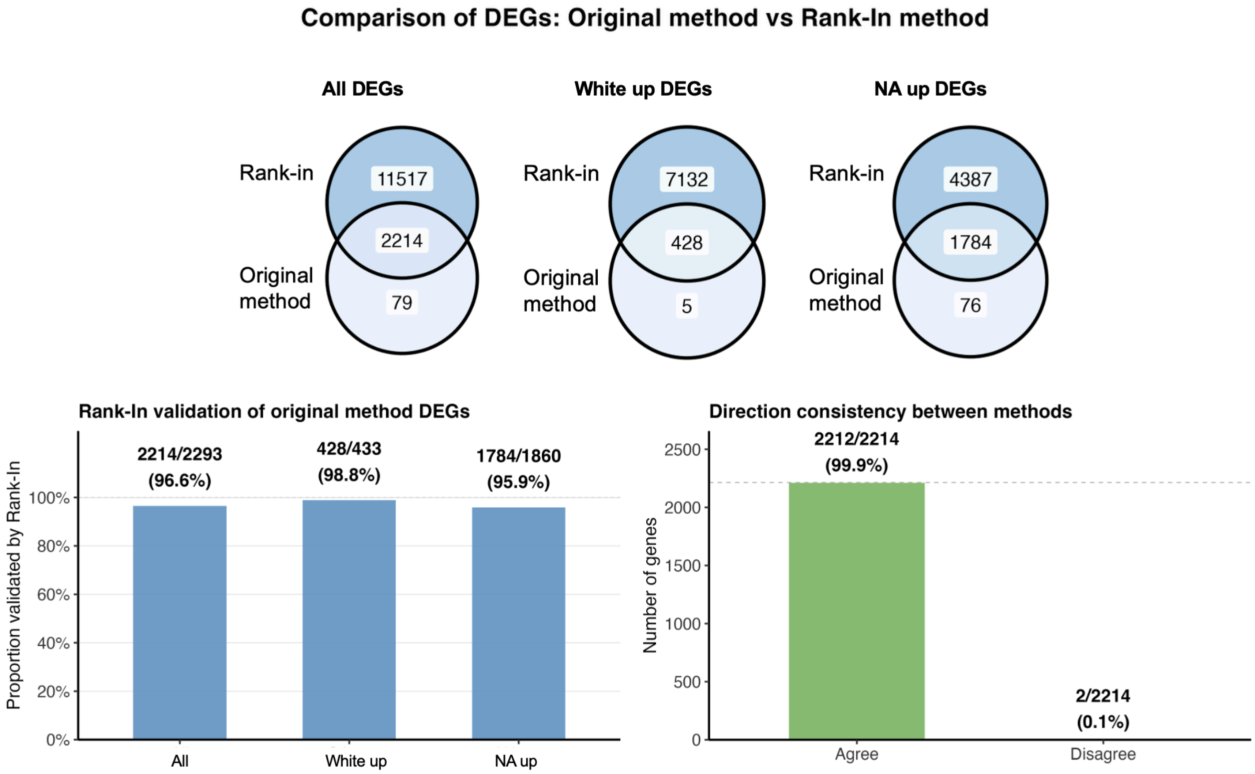

Figure S4: Comparison of DEGs identified by the original method and the Rank-In method. Venn diagrams show the overlap between DEGs detected by the two methods for all DEGs, White-upregulated DEGs, and NA-upregulated DEGs. The original method used FDR<0.01 and |log2FC|>=2, while Rank-In used Wilcoxon rank-based testing with FDR<0.01. Bar plots summarize the proportion of original-method DEGs supported by Rank-In and the consistency of inferred expression direction among overlapping genes, demonstrating strong agreement between the two approaches.
